# Substitution Mapping of the Major Quantitative Trait Loci Controlling Stigma Exsertion Rate from *Oryza glumaepatula*

**DOI:** 10.1186/s12284-020-00397-1

**Published:** 2020-06-09

**Authors:** Quanya Tan, Tuo Zou, Mingmin Zheng, Yuerong Ni, Xin Luan, Xiaohui Li, Weifeng Yang, Zifeng Yang, Haitao Zhu, Ruizhen Zeng, Guifu Liu, Shaokui Wang, Xuelin Fu, Guiquan Zhang

**Affiliations:** Guangdong Provincial Key Laboratory of Plant Molecular Breeding, State Key Laboratory for Conservation and Utilization of Subtropical Agro-Bioresources, South China Agricultural University, Guangzhou, 510642 China

**Keywords:** Stigma exsertion, Outcrossing, Quantitative trait locus, Single-segment substitution line, Wild species, Rice

## Abstract

**Background:**

Stigma exsertion rate (SER) is a key determinant for the outcrossing ability of male sterility lines (MSLs) in hybrid rice seed production. In the process of domestication, the outcrossing ability of cultivated rice varieties decreased, while that of wild *Oryza* species kept strong. Here, we detected the quantitative trait loci (QTLs) controlling SER using a set of single-segment substitution lines (SSSLs) derived from *O. glumaepatula*, a wild *Oryza* species.

**Results:**

Seven QTLs for SER were located on 5 chromosomes. *qSER-1a* and *qSER-1b* were located on chromosome 1. *qSER-3a* and *qSER-3b* were mapped on chromosome 3, and *qSER-3b* was further located at an estimated interval of 898.8 kb by secondary substitution mapping. *qSER-5*, *qSER-9* and *qSER-10* were identified on chromosomes 5, 9 and 10, respectively, and *qSER-9* was delimited to an estimated region of 551.9 kb by secondary substitution mapping. The additive effects of the 7 QTLs ranged from 10.6% to 14.8%, which were higher than those of most loci for SER reported previously.

**Conclusions:**

*qSER-1a* and *qSER-1b* are novel loci for SER on chromosome 1. All of the 7 QTLs have major effects on SER. The major QTLs of SER will help to develop MSLs with strong outcrossing ability.

## Background

Rice is a staple food supplying more than 20% calories for the world population, thus playing a key role in sustaining world food security (Fitzgerald et al. 2009). The success of hybrid rice commercialization has greatly improved the yield of rice (Qian et al. 2016). However, cultivated rice is predominantly self-fertilizing with less than 1% natural cross-pollination (Virmani and Athwal 1973). The outcrossing ability of cultivated rice varieties diminished along with changes in the morphology of rice flowers during the process of domestication (Parmar et al. 1979). Previous studies have shown that the exserted stigma, as a main mating organ, can survive for several days after flowering (Kato and Namai 1987), and then get more opportunities to catch pollens (Xu and Shen 1988; Marathi and Jena 2015). Therefore, the stigma exsertion rate (SER) is a key determinant for the outcrossing ability of male sterility lines (MSLs) in hybrid rice seed production.

Wild *Oryza* species has strong ability of outcrossing. It is reported that perennial wild rice has higher outcrossing than annual types (Oka and Morishima 1967). Cultivated rice tends to have a shorter stigma than the annual wild species. The annual wild species has shorter stigma than their perennial progenitors (Virmani and Athwal 1973; Parmar et al. 1979; Marathi et al. 2015). The wide variability for stigma, style, and their total lengths in wild species of rice might have developed simultaneously during evolution and domestication (Marathi et al. 2015). In the past two decades, many of QTLs controlling SER and related traits have been located on 12 chromosomes of rice genome from various genetic resources (Marathi and Jena 2015; Zhou et al. 2017a; Liu et al. 2019). However, only 2 wild *Oryza* species were used to identify QTLs of SER until now, and *O. rufipogon* was most commonly used. Xiong et al. (1999) reported a QTL for extruding stigma on chromosome 6 using a F_2_ population derived from a cross between *O. sativa*, Aijiao Nante, and *O. rufipogon*, P16. Li et al. (2001) found two QTLs of SER on chromosomes 5 and 8 by a backcross-F_1_ population between *O. rufipogon* and Guichao 2, an elite *indica* rice. Uga et al. (2003a) identified two QTLs for the rate of exserted stigma between the *indica* line Pei-kuh and W1944 of *O. rufipogon*. Huang et al. (2012) found 8 QTLs for SER using the 271 F_2_ lines from the cross between *O. sativa*, Guangluai-4, and *O. rufipogon*. Bakti and Tanaka (2019) detected five QTLs for SER using a F_2_ population generated from a cross between the *japonica* rice cultivar ‘Akidawara’ and ‘W0120’ of *O. rufipogon*. From another wild species, *O. longistaminata*, several QTLs of SER were identified from an introgression line of the wild species in the genetic background of Asian cultivated rice (Li et al. 2010).

*O. glumaepatula* is one of the AA-genome wild *Oryza* species indigenous to Central and South America (Doi et al. 2008). Recently, Stein et al. (2018) elucidated that *O. glumaepatula* is the sister group only of African *O. barthii* and *O. glaberrima* based on the genome sequence alignment. Several hybrid pollen sterility genes between *O. sativa* and *O. glumaepatula* have been identified, such as *S12*, *S22*, *S23*, *S27* and *S28* (Sano 1994; Sobrizal et al. 2000a, b; Sobrizal and Yoshimura 2001, 2002; Sakata et al. 2014; Fang et al. 2019). It was found that the endosperm of *O. glumaepatula* had the high levels of total protein, albumin, and glutelin protein fractions and amino acids, showing the potential to increase the nutritional quality of rice storage protein (Santos et al. 2013). *O. glumaepatula* also showed the rapid internodal elongation potential under partial submergence (Sasayama et al. 2018), as well as its important yield and yield component traits (Zhang et al. 2015; Bhatia et al. 2017). It is important to note that *O. glumaepatula* has a longer stigma length, which is favorable for outcrossing (Marathi et al. 2015).

In order to detect QTLs for traits of agronomic importance in *O. glumaepatula*, 168 SSSLs were developed by using *O. glumaepatula* as donors and Huajingxian74 (HJX74), an *indica* variety of *O. sativa* as recipient. The total length of the substituted segments in the set of SSSLs was 3636.4 cM, covering 12 chromosomes (Zhao et al. 2019). In the present study, we used the set of SSSLs to identify QTLs for SER. Seven of the QTLs were located on 5 chromosomes. The QTLs had higher additive effects. These major QTLs of SER from *O. glumaepatula* will be helpful for developing of MSLs with strong outcrossing ability.

## Results

### SER in the SSSLs Derived from *O*. *glumaepatula*

**Fig. 1:**
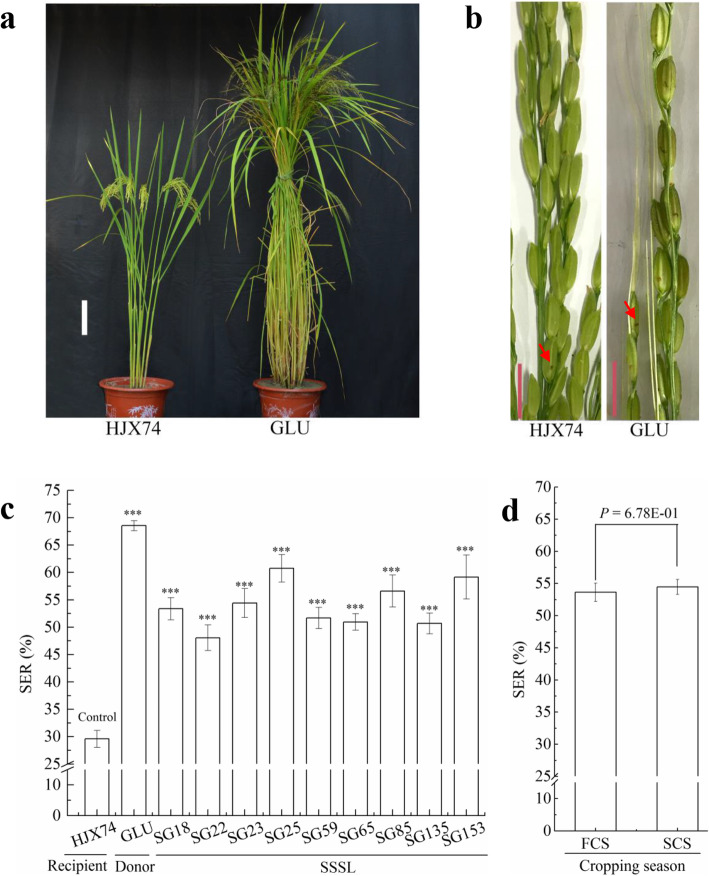
Stigma exsertion of the SSSLs and their parents. **a** Plant type of the parents. Scale bar, 15 cm. **b** Phenotype of exserted stigmas of the parents. The red arrow points to the exserted stigmas. Scale bar, 1 cm. **c** SER in 9 SSSLs and their parents. Data are presented as mean ± S.E. of five cropping seasons. One-way ANOVA, LSD, two-tailed, *** *p*-value≤ 0.001. **d** Comparison of SER between FCS and SCS. Mean ± S.E., two-tailed, two sample Student’s t test. GLU: *Oryza glumaepatula*. SER: stigma exsertion rate. SSSL: single segment substitution line. FCS: first cropping season. SCS: second cropping season

Compared with the HJX74 recipient, *O*. *glumaepatula*, the donor of SSSLs, showed higher SER. On the average of 5 cropping seasons, SER in the donor was 68.6%, while 29.6% in the recipient (Fig. 1a-c and Additional file 1: Table S1). Firstly, a set of 168 SSSLs derived from *O*. *glumaepatula* was investigated for SER and 9 of the SSSLs with higher SER were selected. The 9 SSSLs were then tested their SER for 5 cropping seasons. Compared with the HJX74 recipient, the 9 SSSLs showed higher SER at the *P* ≤ 0.001 level in every cropping season. Average SER of the 9 SSSLs in 5 cropping seasons were from 48.1% to 60.8% with 18.5–31.2% higher than that of the control HJX74 (Fig. 1c and Additional file 1: Table S1). Analysis of variance (ANOVA) of the 9 SSSLs in 5 cropping seasons showed that the *F*-values of lines (9 SSSLs), seasons (5 cropping seasons) and lines by seasons showed significant difference at the *P* ≤ 0.001 level (Additional file 1: Table S2). However, The SER between the first cropping season (FCS) from late February to middle July and the second cropping season (SCS) from late July to middle November showed no significant difference (Fig. 1d).

The chromosomal substituted segments from *O*. *glumaepatula* in the 9 SSSLs were detected by molecular markers. The substituted segments in the 9 SSSLs were detected on chromosomes 1, 3, 5, 9 and 10, respectively. The estimated lengths of substituted segments were from 1467.0 kb to 16,410.5 kb (Additional file 1: Table S3 and Table S4).

Eight agronomic traits in the 9 SSSLs were investigated in two cropping seasons. Compared with HJX74, most of the traits in the SSSLs had no significant difference, except the plant height in SG25, the heading date in SG153, and so on (Additional file 1: Table S5).

### Two QTLs for SER Were Mapped on Chromosome 1

Four SSSLs with high SER, SG18, SG22, SG23 and SG25, carried substituted segments on chromosome 1. SG18 had a substituted segment at the interval from markers RM140 to RM5853 with the estimated length of 10,741.8 kb (Fig. 1c and Additional file 1: Table S4). Therefore, the substituted segment of SG18 carried a QTL for SER, named *qSER-1a*.

**Fig. 2:**
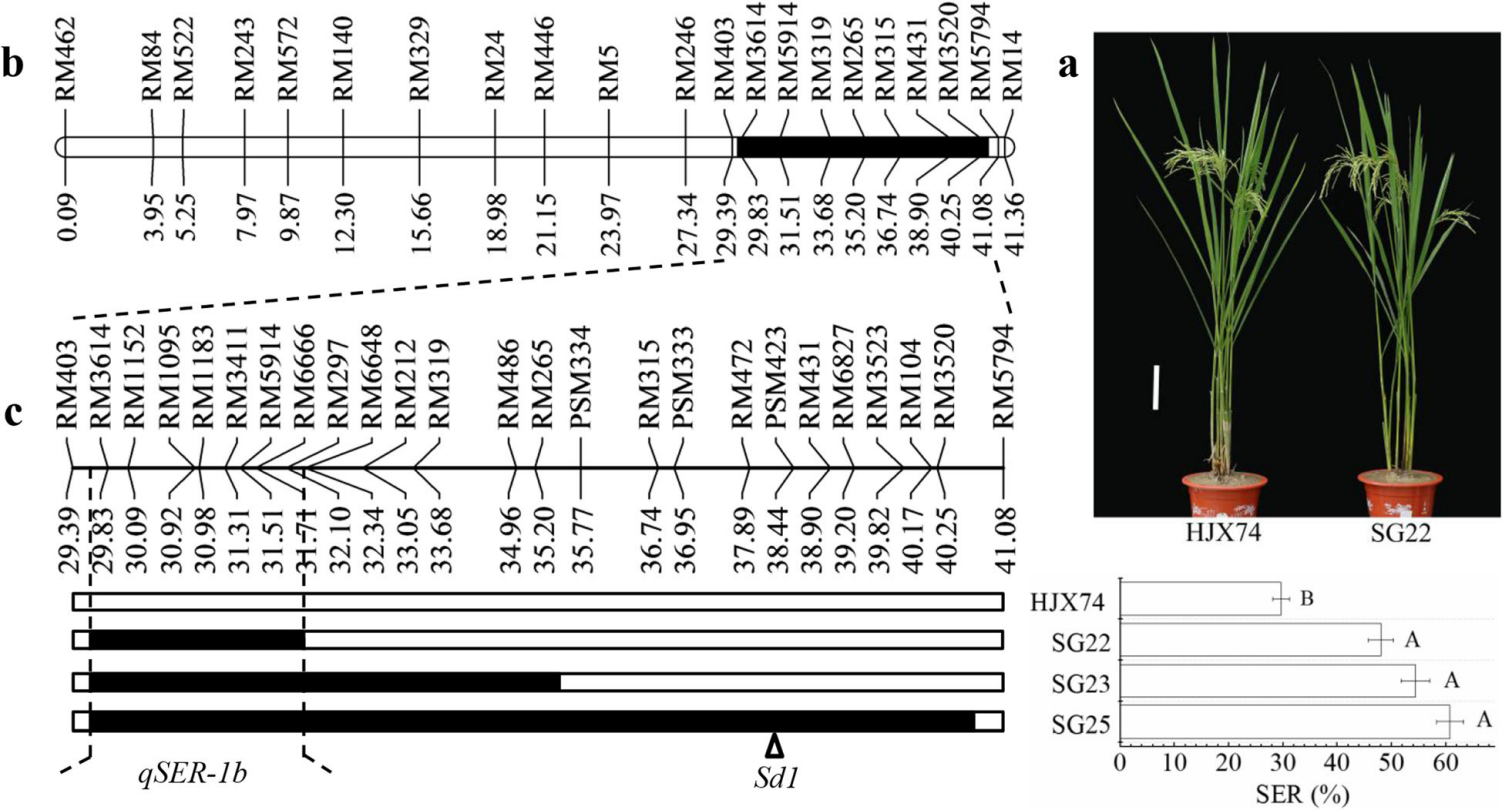
Substitution mapping of *qSER-1b*. **a** Plant type of HJX74 and SG22. Scale bar, 15 cm. **b** The region of substituted segments of three SSSLs, SG22, SG23 and SG25, on chromosome 1. **c** Substitution mapping of *qSER-1b*, showing position of substituted segments and stigma exsertion rate (SER) of the SSSLs with HJX74 as the control. White and black blocks on chromosomes represent genotypes of HJX74 and donor, respectively. Threshold, α = 0.001, one-way ANOVA, Duncan, two-tailed. The triangle represents the locus of *Sd1* (Sasaki et al. 2002)

Three other SSSLs, SG22, SG23 and SG25, carried substituted segments in the region of 29,385.9–41,082.0 kb and the substituted segments overlapped each other. SG22 had the shortest substituted segment with the estimated length of 2611.3 kb from markers RM403 to RM6648. The estimated lengths of substituted segments were 5874.5 kb in SG23, and 11,059.7 kb in SG25. These results indicated that the three SSSLs carried another QTL for SER, named *qSER-1b*, which was located in the substituted segment of SG22 with an estimated interval of 2611.3 kb (Fig. 2 and Additional file 1: Table S4).

Because the *qSER-1b* was linked to *Sd1*, SG25 carried the *Sd1* in the substituted segment from *O*. *glumaepatula* and showed tall in plant height with 160.3 cm in FCS and 135.7 cm in SCS. SG22 and SG23 carried shorter substituted segments without *Sd1* from *O*. *glumaepatula*. Although SG22 and SG23 were semi-dwarf in plant height, the SER had no significant difference with SG25 (Fig. 2 and Additional file 1: Table S5). These results indicated that the SER of *qSER-1b* was not affected by plant height.

### Two QTLs for SER Were Mapped on Chromosome 3

Two SSSLs with high SER, SG59 and SG65, carried substituted segments on chromosome 3. SG59 had a substituted segment at the interval from markers RM282 to InDel3 with the estimated length of 4632.1 kb (Fig. 1c and Additional file 1: Table S4). Therefore, the substituted segment of SG59 carried a QTL for SER, named *qSER-3a*.

**Fig. 3:**
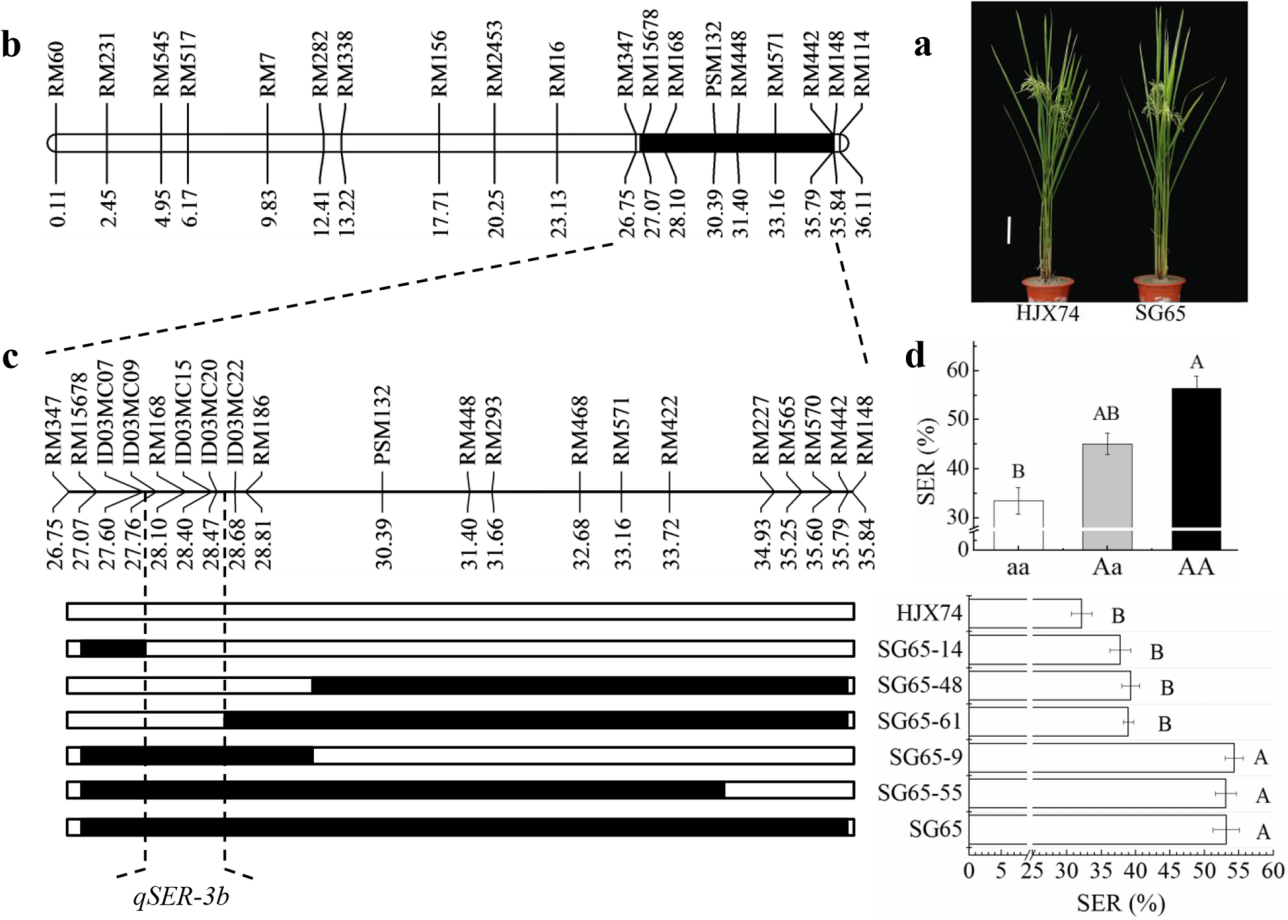
Secondary substituted mapping of *qSER-3b*. **a** Plant type of HJX74 and SG65. Scale bar, 15 cm. **b** The substituted segment of SG65 on chromosome 3. **c** Substitution mapping of *qSER-3b*, showing position of substituted segments and stigma exsertion rate (SER) of the SSSLs with HJX74 as the control. **d** SER effects of three *qSER-3b* genotypes in a F_2_ population. White and black blocks on chromosomes represent genotypes of HJX74 and donor, respectively. aa: Homozygous genotype of HJX74. Aa: Heterozygous genotype of SG65/HJX74. AA: homozygous genotype of SG65. Threshold, α = 0.001, one-way ANOVA, Duncan, two-tailed

The SSSL SG65 carried a substituted segment at the interval of 26,747.5–35,843.1 kb on chromosome 3. The substituted segment was in a different region from that in SG59. Therefore, SG65 had another QTL for SER, *qSER-3b*, in its substituted segment (Fig. 3a-b, Additional file 1: Table S4).

To narrow the interval of *qSER-3b*, 5 secondary SSSLs were developed from a F_2:3_ population derived from the cross of HJX74/SG65. The secondary SSSL, SG65–14, carried a substituted segment in the region from markers RM15678 to ID03MC07 on the left. Two of the secondary SSSLs, SG65–48 and SG65–61, had substituted segments in the regions from PSM132 and ID03MC22 to RM442 on the right, respectively. The three secondary SSSLs showed low SER as HJX74. Other two secondary SSSLs, SG65–9 and SG65–55, carrying substituted segments covering the region from markers ID03MC07 to ID03MC22 showed high SER as SG65. These results indicated that the *qSER-3b* was narrowed to an estimated interval of 898.8 kb between markers ID03MC07 and ID03MC22 (Fig. 3c).

Using the RM168 marker at the *qSER-3b* interval, Chi-square test was carried out in a F_2_ population of 80 individuals. The results revealed that the segregation ratio of three genotypes of the marker fit 1:2:1 (χ^2^ = 0.60 < χ^2^_0.01,2_ = 9.21), and the heterozygous genotype showed incomplete dominance (Fig. 3d).

### Secondary Substitution Mapping of *qSER-9*

**Fig. 4:**
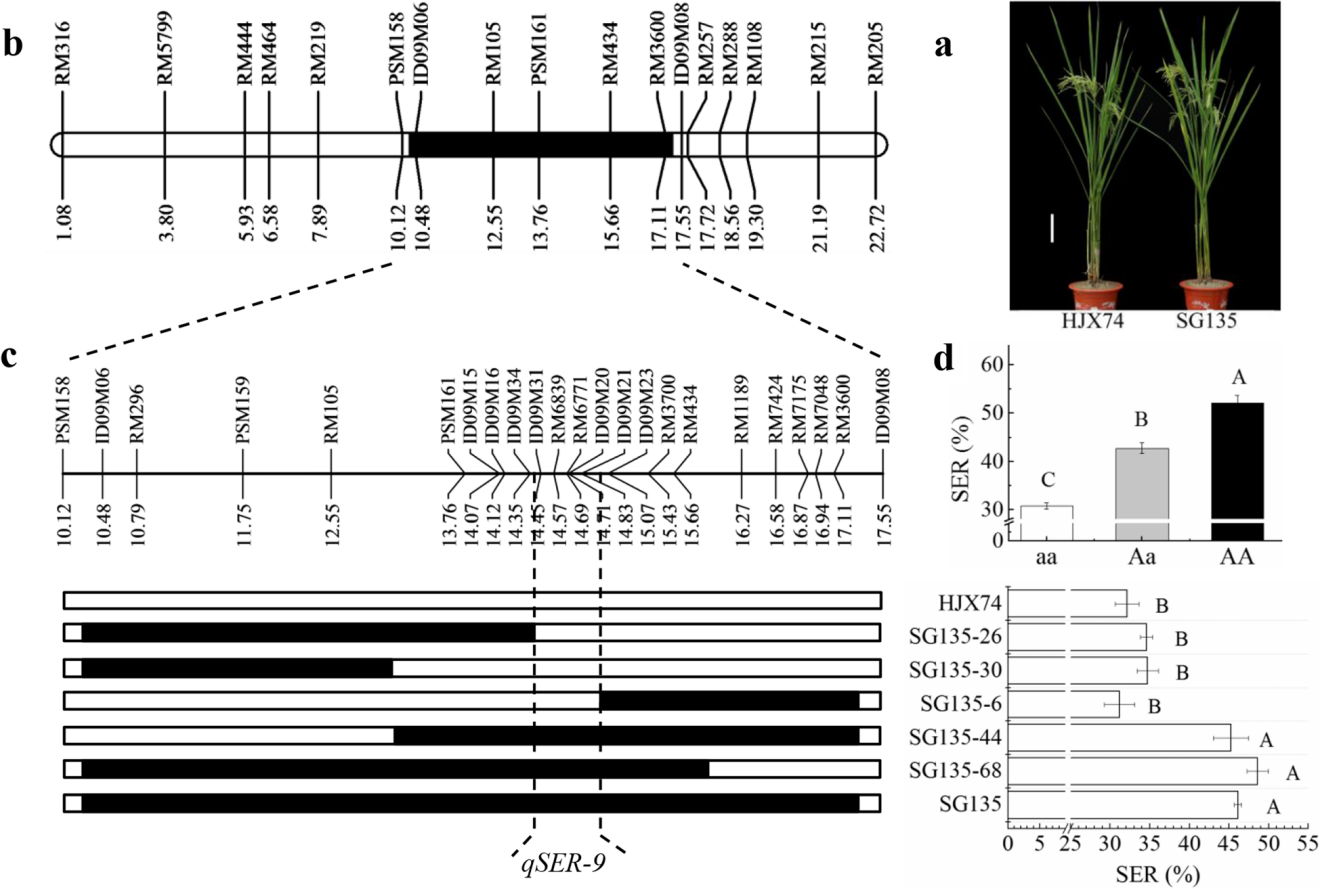
Secondary substitution mapping of *qSER-9*. **a**, Plant type of HJX74 and SG135. Scale bar, 15 cm. **b**, The substituted segment of SG135 on chromosome 9. **c**, Substitution mapping of *qSER-9*, showing position of substituted segments and stigma exsertion rate (SER) of the SSSLs with HJX74 as the control. **d**, SER effects of three *qSER-9* genotypes in a F_2_ population. White and black blocks on chromosomes represent genotypes of HJX74 and donor, respectively. aa: Homozygous genotype of HJX74. Aa: Heterozygous genotype of SG135/HJX74. AA: homozygous genotype of SG135. Threshold, α = 0.001, one-way ANOVA, Duncan, two-tailed

One SSSL, SG135, carried a substituted segment on chromosome 9. Therefore, the substituted segment had a QTL for SER, *qSER-9*. To further map *qSER-9*, 5 secondary SSSLs were developed from a F_2:3_ population from the cross of HJX74/SG135. Two of the secondary SSSLs, SG135–26 and SG135–30, carried respectively substituted segments in the regions from ID09M06 to ID09M34 and RM105 on the left. The secondary SSSL, SG135–6, had a substituted segment in the region from ID09M23 to RM3600 on the right. The three secondary SSSLs showed low SER as HJX74. Two other secondary SSSLs, SG135–44 and SG135–68, carried substituted segments from ID09M34 to ID09M23, and had high SER as SG135. These results indicated that the *qSER-9* was delimited to an interval between ID09M34 and ID09M23 with 551.9 kb estimated length (Fig. 4a-c).

Chi-square test of the RM6839 marker genotypes in a F_2_ population of 80 individuals revealed that the segregation ratio of the three genotypes was 1:2:1 (χ^2^ = 0.48 < χ^2^_0.01,2_ = 9.21) and the heterozygous genotype showed incomplete dominance (Fig. 4d).

### Other QTLs for SER Identified in the SSSLs

One SSSL, SG85, carried a substituted segment from markers RM7444 to RM291 on chromosome 5 with the estimated length of 16,410.5 kb. Therefore, the SSSL had a QTL for SER, *qSER-5*, in the substituted segment (Fig. 1c and Additional file 1: Table S4).

Another SSSL, SG153, carried a substituted segment from markers RM484 to RM25886 on chromosome 10 with the estimated length of 1467.0 kb and had a QTL for SER, *qSER-10*, in the substituted segment (Fig. 1c and Additional file 1: Table S4).

**Fig. 5:**
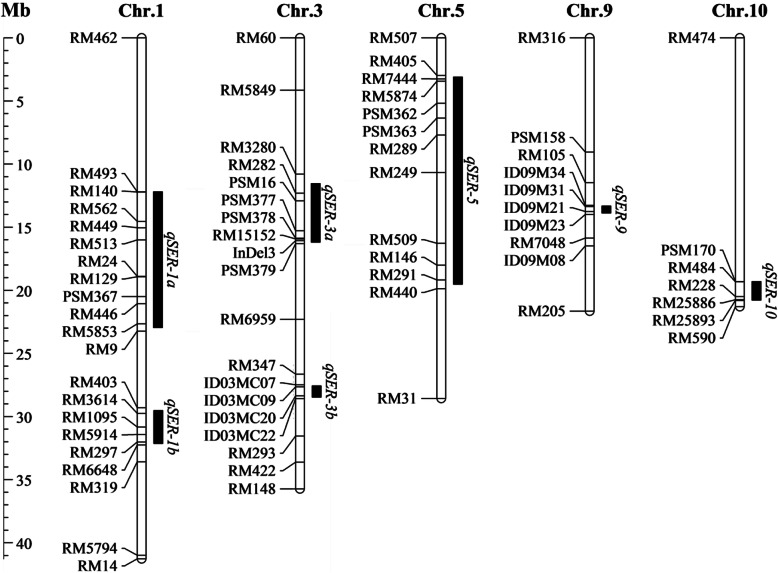
Chromosomal locations of the seven QTLs for SER in the SSSLs. Black bars on the right of each chromosome are the estimated intervals of QTLs with their names on the right. *Chr.* chromosome, *Mb* megabase

Summarily, a total of 7 QTLs for SER was mapped on 5 chromosomes. Among of them, chromosomes 1 and 3 each carried two of the QTLs, and chromosomes 5, 9 and 10 each carried one of the QTLs (Fig. 5).

### The Additive Effects of QTLs for SER Identified in the SSSLs

The additive effects of the 7 QTLs for SER, *qSER-1a*, *qSER-1b*, *qSER-3a*, *qSER-3b*, *qSER-5*, *qSER-9* and *qSER-10*, ranged from 10.6% to 14.8%. Among of them, *qSER-10* had the highest additive effect of 14.8% (Table 1).

**Table 1:** Additive effects of QTLs for stigma exsertion rate detected in the SSSLs

QTL	Chr.	Interval (kb)	Estimated length (kb)	Maximum length (kb)	*P* value	A (%)
*qSER-1a*	1	12,291.6–23,033.4	10,741.8	11,044.9	1.8E-09	11.9 ± 0.8
*qSER-1b*	1	29,608.2–32,219.6	2611.3	2952.5	1.2E-12	12.4 ± 1.8
*qSER-3a*	3	11,656.8–16,288.9	4632.1	5502.7	1.2E-08	11.1 ± 1.1
*qSER-3b*	3	27,677.1–28,575.9	898.8	1087.9	2.7E-08	10.7 ± 1.2
*qSER-5*	5	3216.5–19,626.9	16,410.5	16,901.8	4.9E-11	13.5 ± 1.6
*qSER-9*	9	14,398.5–14,950.4	551.9	718.7	3.6E-08	10.6 ± 1.0
*qSER-10*	10	21,132.3–22,599.3	1467.0	1504.7	2.9E-12	14.8 ± 2.0

*A* additive effect, *A* was represented as mean ± S.E. in five cropping seasons. *P* value indicates significant degree of SER between SSSL(s) and HJX74, determined by one-way ANOVA, LSD, two-tailed

## Discussion

In the past two decades, many of QTLs controlling stigma exsertion were identified in rice genome. The QTLs were distributed across all 12 chromosomes (Marathi and Jena 2015; Zhou et al. 2017a; Liu et al. 2019). Among of them, a limited number of QTLs for SER were identified from the wild *Oryza* species, *O. rufipogon* (Xiong et al. 1999; Li et al. 2001; Uga et al. 2003a; Huang et al. 2012; Bakti and Tanaka 2019) and *O. longistaminata* (Li et al. 2010). In the present study, we detected 7 QTLs of SER on 5 chromosomes using a set of SSSLs derived from *O. glumaepatula* (Fig. 5). On chromosome 1, *qSER-1a* and *qSER-1b* are novel loci, in which region no other QTLs for SER were reported previously. On chromosome 3, *qSER-3a* was located in the QTL cluster of *qES3* (Miyata et al. 2007), *PES-3* (Yue et al. 2009), *qSPE3* (Feng et al. 2010) and *qSSE3* (Li et al. 2014a). *qSER-3b* was mapped in the region locating of *qTSE-3b* (Li et al. 2014b) and *qSER-3.2* (Xu et al. 2019). On chromosome 5, the region of *qSER-5* covered those of *qPEST-5* (Li et al. 2001), *qPES-5* (Deng et al. 2010), *qTSE-5a* (Li et al. 2014b), *TSE* (Zhou et al. 2017a) and *qSER-5.1* (Xu et al. 2019). On chromosome 9, the *qSER-9* region overlapped partly with the segment of *qPES-9* from T821B, an introgression line from *O. longistaminata* (Li et al. 2010). In addition, the *qSER-10* interval on chromosome 10 was overlapped with the region of *qDSE-10* (Li et al. 2014a). Although most of the loci identified in this study were located in the same regions as the previously reported loci, it is worth noting that the additive effects of the loci identified from *O*. *glumaepatula* were greater. The additive effects of the seven loci identified in this study were between 10.6% and 14.8% (Table 1), while those of the previously reported loci were usually less than 8.0%. It indicated that the QTLs from *O*. *glumaepatula* had major effects on SER, which will be favorable for molecular breeding of MSLs with greater ability for outcrossing.

During the process of domestication, cultivated rice has already lost some traits of natural outcrossing (Parmar et al. 1979). Wild *Oryza* species have a strong outcrossing ability due to their larger stigma, longer style, greater exsertion of the stigma, and longer periods of spikelet opening (Marathi et al. 2015; Marathi and Jena 2015). Previous studies revealed dominant differences for SER and floral traits between cultivated rice and wild rice (Virmani and Athwal 1973; Uga et al. 2003b). It was found that *O. glumaepatula* had longer of stigma length than cultivated rice (Marathi et al. 2015). In the present study, we identified 7 QTLs for SER with major effects from the SSSLs derived from *O. glumaepatula* (Table 1). It indicated that the favorable alleles for outcrossing traits, which had been lost in cultivated rice, could be mined in wild *Oryza* species.

It is well documented that the *GS3* gene not only determines seed length but also exerts a pleiotropic effect on stigma length and exsertion, such that plants with the *gs3* allele often have long kernels and higher SER (Miyata et al. 2007; Zhou et al. 2017a; Xu et al. 2019). It was found that the introduction of the *GS3* gene into a *japonica* rice (with the *gs3* allele) significantly reduced stigma exsertion in transgene-positive plants, suggesting that *GS3* influences stigma exsertion (Takano-Kai et al. 2011). However, there are rice accessions with very low SER that also carry the *gs3* allele, suggesting that the beneficial effect of *gs3* on stigma exsertion is dependent on genetic background (Xu et al. 2019). Zhou et al. (2017a) found that rice accessions with the *GW5gs3* combination had the highest stigma exsertion but still much lower than that of wild rice, indicating other loci contributions in rice domestication. In our research, HJX74 with *GW5GS3* genotype showed low SER, while the SSSLs carrying QTLs of SER on its substituted segments showed high SER (Fig. 1c and Fig. 5). These results indicated that the high SER in the SSSLs was independent on the *gs3* gene. Furthermore, most of the SSSLs showed no significant difference in grain size with HJX74 (Additional file 1: Table S5). Therefore, the high SER in the SSSLs wasn’t significantly affected by grain size.

In the past two decades, we have constructed a SSSL library by the using of HJX74 as the recipient and 43 accessions from 7 species, including 5 wild *Oryza* species of AA genome as donors (Zhang et al. 2004; Xi et al. 2006; Zhang 2019). The SSSLs have been used to map QTLs (Zhang et al. 2012; Yang et al. 2016; Zhou et al. 2017b), to clone genes (Wang et al. 2012; Sui et al. 2019), and to analyze allelic variation (Teng et al. 2012; Cai et al. 2013). Based on the SSSL library, we have developed a platform of breeding by design for cytoplasmic male sterility (CMS) lines and restorer lines, and a series of CMS lines and restorer lines were developed (Dai et al. 2015, 2016; Luan et al. 2019). The QTLs for SER detected in the present study will be used to develop CMS lines with strong outcrossing ability under the platform of SSSL library. Because SER was controlled by multiple QTLs and each QTL had only partial effect (Fig. 1c and Table 1), it is necessary to pyramid multiple QTLs to develop CMS lines with strong crossing ability. Although the effect of QTLs was affected by environments (Additional file 1: Table S2), the SER of plants had no significant difference between FCS and SCS (Fig. 1d). Therefore, CMS lines pyramiding the QTLs of SER will be applied to practical hybrid seed production both in FCS and in SCS. In addition, enhancing of outcrossing ability will be helpful to raise grain yield in hybrid rice. It is worth noting that the heterozygous genotype of the QTLs for SER showed incomplete dominance (Fig. 3d and Fig. 4d). Therefore, pyramiding of the QTLs for SER both in CMS lines and in restorer lines will help to raise the seed set of hybrid rice.

## Conclusion

Using the SSSLs derived from *O. glumaepatula*, seven major QTLs controlling SER, *qSER-1a*, *qSER-1b*, *qSER-3a*, *qSER-3b*, *qSER-5*, *qSER-9* and *qSER-10*, were located on 5 chromosomes by substitution mapping. *qSER-1a* and *qSER-1b* are novel loci for SER, in which regions no other QTLs of SER were reported previously. Compared with the previously reported loci, the 7 QTLs have major additive effects. The major QTLs will be favorable for molecular breeding of MSLs with strong outcrossing ability. The favorable alleles for outcrossing traits, which were lost in cultivated rice, could be mined in the wild *Oryza* species.

## Methods

### Materials and Filed Experiment

The set of SSSLs derived from *O. glumaepatula* (IRGC104387), an accession of the wild species indigenous to Brazil (Zhao et al. 2019) and their parents HJX74 and *O. glumaepatula* were grown in the paddy fields at the experimental farm of South China Agricultural University in Guangzhou (23°07′N, 113°15′E), China. The materials were grown in 5 cropping seasons from 2016 to 2018, two cropping seasons per year, FCS and SCS. Single-seedling transplanting was applied in field experiment. Each plot had four rows with 10 single plants per row. Standard cultivation practices and controlling of diseases and insect pests were followed the typical methods in South China.

### Molecular Markers and PCR Protocol

SSR markers labeled “RM” were selected from online resources (https://archive.gramene.org/markers/). The “PSM” and “Indel” markers were designed using the software of Primer Premier 5.0 (Lalitha 2000). DNA was extracted from the fresh leaves of each plant referencing a reported method (Murray and Thompson 1980). The PCR products were amplified and analyzed on 6% denatured polyacrylamide gel, and detected using the silver staining using the methods described by Fang et al. (2019).

### Phenotyping of Traits and Statistical Analysis

The stigma exsertion (SE) was subdivided into single stigma exsertion (SSE) and dual stigma exsertion (DSE). The SER refers to total stigma exsertion rate, including single stigma exsertion rate and dual stigma exsertion rate. For investigating SER, 8–10 main panicles were sampled from plants in 1–2 days after flourishing florescence of florets. The spikelet numbers of SSE and DSE per panicle were counted based on visual exserted stigmas (Xu et al. 2019). Grain traits were measured by using the YTS rice phenotypic facility (Yang et al. 2014). Differences of traits were determined using one-way ANOVA. SPSS 23.0 and OriginPro 9.0 (https://www.originlab.com) were applied to analyze the experimental data.

### Mapping of QTLs

The lengths of substituted segments of SSSLs were calculated by the previous method (Xi et al. 2006; He et al. 2017; Zhao et al. 2019). The minimum length (L_min_) of a substituted segment refers to the length between two markers of donor genotype at the end of the substitution segment. The maximum length (L_max_) refers to the length between two markers of background genotype flanking the substitution segment. The estimated length (L_est_) = (L_min_ + L_max_)/2. The QTLs for SER were mapped by the substitution mapping method (Eshed and Zamir 1995; Wissuwa et al. 2002). Linkage maps of markers were drawn by using MapChart2.2 (https://www.wur.nl/en/show/Mapchart.htm). QTLs were named by the method of McCouch et al. (1997).

## Supplementary information

**Additional file 1: Table S1.** Stigma exsertion rate of the 9 SSSLs in 5 cropping seasons. **Table S2.** Analysis of variance based on a fixed-effect model of the 9 SSSLs in 5 cropping seasons. **Table S3.** Developed markers used to detect the substituted segments of the SSSLs. **Table S4.** Substituted segments detected by markers in the SSSLs. **Table S5.** Phenotypes of agronomic traits in the SSSLs.

## Data Availability

All data generated or analyzed during this study are included in this published article and its additional information files.

## Abbreviations

CMS: Cytoplasmic male sterility
DSE: Dual stigma exsertion
FCS: First cropping season
MSL: Male sterility line
QTL: Quantitative trait locus
SCS: Second cropping season
SER: Stigma exsertion rate
SSE: Single stigma exsertion
SSSL: Single-segment substitution line
